# Correlates of physical activity and sitting time in adults with type 2 diabetes attending primary health care in Oman

**DOI:** 10.1186/s12889-017-4643-7

**Published:** 2017-08-01

**Authors:** Thamra S. Alghafri, Saud M. Alharthi, Yahya Al-farsi, Elaine Bannerman, Angela M. Craigie, Annie S. Anderson

**Affiliations:** 1 Health Services, Ministry of Health, PO Box 2723, Postal Code 112 Muscat, Oman; 2 Department of Family Medicine and Public Health, College of Medicine and Health Sciences, Sultan Qaboos University, Muscat, Oman; 3 Centre for Public Health Nutrition Research, University of Dundee, Ninewells Hospital and Medical School, Dundee, UK

**Keywords:** Physical activity, Type 2 diabetes, Primary health care, Correlates, Sitting time, Consultations, Oman

## Abstract

**Background:**

Despite evidence of the benefits of physical activity in the management of type 2 diabetes, it is poorly addressed in diabetes care. This study aimed to identify the prevalence and correlates of meeting ≥600MET-min/wk. (150 min/wk) of physical activity and sitting time in adults with type 2 diabetes in Oman. Approaches to encourage physical activity in diabetes care were explored.

**Methods:**

A cross-sectional study using the Global Physical Activity Questionnaire was conducted in 17 randomly selected primary health centres in Muscat. Clinical data including co-morbidities were extracted from the health information system. Questions on physical activity preferences and approaches were included. Patients were approached if they were ≥18 years, and had been registered in the diabetes clinic for >2 years.

**Results:**

The questionnaire was completed by 305 people (females 57% and males 43%). Mean age (SD) was 57 (10.8) years and mean BMI (SD) was 31.0 (6.0) kg/m^2^. Duration of diabetes ranged from 2 to 25 (mean 7.6) years. Hypertension (71%) and dyslipidaemia (62%) were common comorbidities. Most (58.4%) had an HbA1c ≥7% indicating poor glycaemic control (55% in males vs 61% in females).

Physical activity recommendations were met by 21.6% of the participants, mainly through leisure activities. Odds of meeting the recommendations were significantly higher in males (OR 4.8, 95% CI 2.5–9.1), individuals ≤57 years (OR 3.0, 95% CI 1.6–5.9), those at active self-reported stages of change for physical activity (OR 2.2, 95% CI 1.2–4.1) and those reporting no barriers to performing physical activity (OR 2.7, 95% CI 1.4–4.9).

Median (25th, 75th percentiles) sitting time was 705 (600, 780) min/d. Older age (>57 years) was associated with longer sitting time (>705 min/d) (OR 2.8, 95% CI 1.7–4.6).

Preferred methods to support physical activity in routine diabetes care were consultations (38%), structured physical activity sessions (13.4%) and referrals to physical activity facilities (5.6%) delivered by a variety of health care providers.

**Conclusions:**

The results suggest that intervention strategies should take account of gender, age, opportunities within daily life to promote active behaviour and readiness to change. Offering physical activity consultations is of interest to this study population, thus development and evaluation of interventions are warranted.

**Electronic supplementary material:**

The online version of this article (doi:10.1186/s12889-017-4643-7) contains supplementary material, which is available to authorized users.

## Background

In 2013, the International Diabetes Federation (IDF) estimated that 8.3% of the global population have diabetes (382 million) of which 90% have type 2 diabetes (T2D) [1]. The number of people with diabetes is expected to increase by 55% (to 592 million) in 2035. In countries of the Middle East and North Africa (MENA) region, the negative impact of diabetes on health care system expenditures, population productivity and quality of life is of great concern, especially in the Arab countries of the Gulf Cooperation Council (GCC) where prevalence of diabetes is high [2]. Saudi Arabia, Kuwait and Qatar were reported by the IDF in 2013 to be within the top 10 countries with the highest prevalence of diabetes (24.0%, 23.1% and 22.9% respectively) [1, 3]. Oman, similar to the other high income GCC countries, has gone through rapid economic development leading to consumption of energy dense diets and sedentary lifestyles [4]. In Oman, diabetes prevalence increased from 8.3% in 1991 to 12.3% in 2008 and the current estimate reported by IDF is 14.2% [1, 5]. Management of diabetes in Oman and other GCC countries is a public health concern as the countries of the MENA region are estimated to have a 96% increase in number of people with diabetes by 2035 [1].

Physical inactivity is estimated as being the principal cause for 27% of diabetes, and 30% of ischemic heart disease. Similarly, greater sitting time is considered an independent risk factor for diabetes, cardiovascular disease, and all-cause mortality [6]. Sitting more than 8 h/day leads to increase risk of all-cause mortality even among individuals achieving the recommended 150 min/wk. of physical activity (PA). As such, addressing low levels of activity and sedentary behaviours are required to reverse this trajectory.

Evidence for the positive effects of PA on individuals with diabetes is consistent. PA for 150 min a week has been shown to increase insulin sensitivity, lower blood sugar levels, reduce body fat and improve general health [7]. However, in western countries, mainly United States of America (USA), over 60% of patients with diabetes don’t meet the recommended levels of PA [8]. This is similar to the proportion of adults from the general population in Arabic countries including the GCC countries where it was reported that only 39.0% to 42.1% of men and 26.3% to 28.4% of women meet recommended levels of PA [9]. However, in Oman, reported activity levels are lower. The 2008 Oman World Health Survey, reported that only 15.0% of patients with T2D meet PA recommendations of 150 min a week of moderate to vigorous activity [5]. In Sur (north-east coast of Oman), correlates for physical inactivity in the general population (*n* = 1373) were reported using the Global Physical Activity Questionnaire (GPAQ) [10]. Inactivity (<600MET-min/wk) in men increased with age and Body Mass Index (BMI); with every unit of BMI increase the travel inactivity increased by 6.0%. Higher odds of leisure inactivity were seen in males with lower levels of education, individuals who were not employed and married individuals. Women aged ≥40 years had more than double the odds of being inactive compared with the youngest in travel inactivity and the odds of leisure inactivity were approximately 1.8-fold higher in employed women versus unemployed. In the same study, reported mean sitting time was 120 min/d [11] which is considerably low compared with mean sitting time of >270 min/d in Saudi Arabia [12] and >220 min/d in Kuwait [13]. Nonetheless, evidence on sitting time patterns in sub-groups of populations (including people with diabetes) in the Arab world is scarce, but is a potential area of concern.

Socio-demographic, psychosocial and environmental factors have been reported to be associated with PA patterns in populations with T2D, but these associations vary widely across studies [14–16]. Consolidated evidence on levels and correlates of PA and sitting time in Arab countries (especially the countries of the GCC) has never been explored despite the socio-cultural and environmental differences, which are likely to influence behaviour and activity levels.

The present study aimed to collect preliminary data to inform a PA intervention design in diabetes primary care. To do so, the study aimed to describe the PA patterns of adults with T2D and examine the sociodemographic factors, physiological factors and perceptions of PA associated with meeting the World Health Organization’s recommended PA levels of ≥600MET-min/wk. [10], and prolonged sitting time. Secondly, it aimed to identifiy views for integrating PA in routine diabetes care within local primary health care (PHC) setting.

## Methods

A cross-sectional interview based survey was conducted during April and May 2015 in Muscat, Oman using the GPAQ [17]. Omani patients with T2D attending their routine diabetes clinics in 17 randomly selected centres of PHC were invited to participate. Inclusion criteria were: age ≥ 18 years, under diabetes clinic care for more than 2 years and ability to provide informed consent. For illiterate participants, informed consent was taken from their spouse, son, daughter or other close family member after verbal discussions and approvals from the concerned participants. Participants with type 1 diabetes, newly diagnosed patients (due to incomplete data in the electronic health information system), those who had difficulty in performing any PA (due to physical disabilities), those with a history of myocardial infarction of <6 months, or with multiple organ failure, were excluded.

### Sample size

The sample size was calculated using an estimated 15% prevalence of meeting the PA recommendations in patients with diabetes as reported previously in the 2008 Oman World Health Survey [18]. For 95% confidence limits, a response rate of 80%, and a precision of 20%, the calculated sample size was 305 participants over Muscat, the capital of Oman and where 50% of the Omani population live. Muscat region has a total of six urban willayats “districts” (Seeb, Bausher, Amirat, Qryat, Muttrah and Muscat) which were all included in the study.

### Data collection tool

A multi-section questionnaire was designed to collect the following data through interviewing the participants:

#### Socio-demographic data

Multiple choice questions on gender, willayat, age, marital status, education, household income and work status.

#### Perceptions on stages and status of physical activity

A scale developed by Martin et al. [19] was used to report stages of PA based on the trans-theoretical theory of behaviour change. Subjects who were participating in moderate physical exercise five or more times per week or in vigorous exercise three to five times per week longer than six consecutive months were categorized to “Maintenance stage” or if it was less than 6 months to “Action stage”. “Preparation stage” was for subjects who were thinking about starting exercise or walk in the near future, or who were doing vigorous exercise less than three times per week, or moderate physical exercise less than five times per week. Contemplation stage “getting ready” was for subjects who were thinking about starting PA including walking in the next 6 months. Subjects who were not thinking about starting any PA in the near future were categorized as Pre-contemplation stage “not ready”. In addition, participants were asked to answer “yes” or “no” to the questions “have you received any PA advice in the past six months within their diabetes care by the diabetes care team in the health centre?”, “do you think you are performing sufficient PA?”, and “do you perceive any barrier to performing PA?”. Participants were further asked to describe any perceived barriers to their participation in PA. Detailed results for Barriers to performing PA in this population is presented elsewhere. [20].

#### Levels of physical activity and sitting time

The 16 item GPAQ was developed by WHO for PA surveillance and is used in more than 100 countries globally [17]. It estimates PA (intensity, duration, and frequency) performed in three domains - work (paid and unpaid including housework), travel (walking and cycling) and leisure, which includes total sitting time. PA was estimated by calculating energy expenditure using the Metabolic Equivalent (MET), the ratio of specific PA metabolic rates compared with the resting metabolic rate (one MET is equivalent to the energy cost of sitting quietly, kcal/kg/h). Total MET-min/d was calculated for each domain by first multiplying MET values by reported minutes (moderate-intensity and transport activity assigned 4MET values and vigorous-intensity activities assigned 8MET values), then adding the total MET-min of vigorous and moderate intensity activities performed [17].

Estimated weekly PA levels (including activity for work, during travel and leisure time), were compared against WHO PA recommendations of 150 min of moderate-intensity PA or 75 min of vigorous-intensity PA per week (which equates to an equivalent combination of moderate- and vigorous-intensity PA achieving at least 600 MET-min/wk. [10].

A single open-ended question regarding total sitting time is included in GPAQ as “Over the past seven days, how much time did you spend sitting or reclining on a typical day?” Subjects were requested to estimate their sitting time in minutes per day.

Additionally, information on physiological data (health status and anthropometric measures) were collected from the electronic health information system coinciding with diabetes including duration of diabetes, BMI, medication, blood pressure, lipid profile, and presence of any comorbidities defined as cardiovascular, hyperlipidemia, thyroid abnormalities, renal, eye, musculoskeletal, or any other recorded condition in the system.

Participants were also asked to select their preferred PA (from a list of walking, jogging, running, swimming, football, and others to be specified), and suggest PA intervention components to be integrated within routine diabetes clinics in PHC and who it should be delivered by in the health centre.

### Training

Health care staff were recruited for data collection and received training on conducting the interview. Before full-scale sampling began a pre-test with 25 participants from a population outside the sampled health centres was undertaken to evaluate face validity, ease of questioning and the length of time to administer the questionnaire. Study related data collection procedures, dynamics and tool were all modified accordingly.

### Ethical approval

Potential participants were invited to be interviewed for the survey when they entered the clinic or waited for clinical staff. All participants were provided with written information and provided informed consent prior to commencement of the interview. For illiterate individuals, consents to participate in this study were provided by their accompanying support member (spouse, son or daughter) who could at least read and write.

### Data collection, and entry

Quality of entered data was cross-checked by a nurse trained in quality assurance using check lists specific to the study in a sample of 10% of questionnaires selected at random.

Data entry, cross-checking and cleaning was done through Epi Info™ 7. Entered data was transferred to SPSS v21 for analysis according to GPAQ protocol [17].

### Statistical analyses

Descriptive statistics were expressed as mean (SD), median (25th, 75th percentiles) or percentages and number of active cases for the total study population as appropriate. All statistical tests were two sided and at a significance level of 0.05. Bivariate relationships between the dependent variable of meeting WHO PA recommendations and the independent variables, namely socio-demographic (gender, region, age, marital status, education, income, and work status), physiological (BMI, medication, duration of diabetes, blood pressure, lipid profile, and reporting comorbidities), and self-reported perceptions of PA (self-reported levels of PA, receiving PA advice, self-perceptions performing sufficient PA/wk., reporting barriers to leisure PA), were tested by chi-square analyses. Potentially significant associations with *P* values <0.05 were further analyzed using binary logistic regression. The categories of several variables were collapsed to ensure sufficient power for the regression models and adequate numbers in all categories. For example, age was dichotomised using mean value (in years) of ≤57 vs >57, married vs unmarried, educated vs uneducated, income <500 or ≥500, and active vs inactive self-reported stages of PA. Backward stepwise elimination was utilised to select the best model with significant variables that could best predict the behaviour of meeting PA recommendations. Initially all potential variables with significant *P*-values on chi-square test were included in the model. Variables with *P* values >0.05 were dropped one by one until a significant model with the largest adjusted R^2^ criterion was reached and hence deemed to be the best model fit. The odds ratios were calculated for socio-demographic variables (against the reference categories of female, subjects aged >57 years, currently married, educated, with income of ≥500 Omani rials, and employed), physiological variables (due to more participants numbers, the reference category was reporting existing co-morbidities), and self-reported perceptions of PA (against the reference categories of reported inactive stages of PA (“not ready” and “getting ready”) and reporting performing sufficient PA/wk. and reporting barriers to leisure PA).

Mann-Whitney U non parametric test was used to identify the association of sitting time with meeting PA recommendation. Whilst the literature is inconsistent on average, low and high sitting times for this population, sitting time was dichotomised around the median value (≤705 min/d and >705 min/d) to allow the determination of any correlates associated with this behaviour.

Preferences for PA, and the PA delivery components of interest to adult patients with T2D in health centres are reported as proportions of the population.

## Results

### Socio-demographic

During the study duration, 312 patients were invited to participate and 305 completed the questionnaire (98%), with slightly greater proportion of females than males (57.4% vs 42.6%). The majority of the sample was from Seeb willayat (41.7%), a highly populated region in Muscat. Mean (SD) age was 57 (10.8) years with more than two-thirds being married (78.8%) and almost half indicating they ‘don’t read or write’ (48.9%). Thirty nine percent of subjects reported house hold income of <500 Omani rials. Most females were housewives (77.0%). It was noted that more males than females were government employees (14.6% and 2.9% respectively) (Table 1). Meeting the PA recommendations was more common in males *P* < 0.001, unmarried individuals *P* = 0.004, those who completed higher education *P* = 0.030, and had an income of 500- < 1000 Omani rials *P* = 0.008, government employees *P* < 0.001.

**Table 1:** Sample characteristics (socio-demographic variables) and prevalence of meeting physical activity recommendations

Sample characteristics	Total sample	Meeting physical activity recommendations	*P*-value
	*n* = 305(%)	*n* = 66 (21.6%)	
Gender			<0.001*
Male	130 (43)	45 (35)	
Female	175 (57)	21 (12)	
Willayat			0.060
Alamirat	42 (14)	4 (10)	
Bousher	37 (12)	3 (8)	
Muscat	22 (7)	3 (14)	
Muttrah	63 (21)	23 (37)	
Quryat	14 (4)	2 (14)	
Aseeb	127 (42)	31 (24)	
Age categories (years)			0.050
< 40	21 (7)	10 (48)	
40–49	54 (18)	14 (26)	
50–59	98 (32)	24 (24)	
60–69	92 (30)	15 (16)	
≥ 70	40 (13)	3 (8)	
Marital status			0.004*
Unmarried	8 (3)	3 (38)	
Currently married	240 (79)	57 (24)	
Separated/divorced	20 (6)	5 (25)	
Widowed	37 (12)	1 (3)	
Education			0.030*
Don’t read or write	149 (49)	18 (12)	
Less than primary	49 (16)	8 (16)	
Primary completed	28 (9)	8 (29)	
Preparatory completed	27 (9)	13 (48)	
Secondary completed	30 (10)	11 (37)	
College completed	10 (3)	4 (40)	
Higher education completed	11 (4)	5 (45)	
Income (Omani Rials)			0.008*
< 500	120 (39)	22 (18)	
500- < 1000	100 (33)	35 (35)	
1000- < 1500	17 (6)	4 (24)	
≥ 1500	14 (5)	3 (21)	
No answer	54 (17)	2 (4)	
Employment		0.02	<0.001*
Government employee	24 (8)	12 (50)	
Non-government employee	35 (11)	13 (37)	
Self-employed	12 (4)	4 (33)	
Retired	77 (25)	19 (25)	
Unemployed	157 (52)	16 (10)	

*significant *p* < 0.05 based on chi-square analysis

### Physiological

Duration of diabetes extended from 2 to 25 [mean (SD) 7.59 (4.7) years, and median (range) 6 (23) years]. Eighty-nine percent of the sample were overweight or obese, with half classed as obese (50.2%) [mean (SD) BMI 30.96 (6.01) kg/m^2^]. More females were classed as obese compared to males (59.4% vs 37.7%), however, a greater proportion of males were overweight compared to females (44.6% vs 34.3%). The majority of subjects were on oral hypoglycaemic drugs compared to diet only (85.2% vs 14.8%) with a quarter using insulin in addition to the oral drugs (24.6%). Hypertension and dyslipidaemia were the most common comorbidities (71.1% and 62.0% respectively) (Table 2).

**Table 2:** Sample characteristics (physiological variables) and prevalence of meeting physical activity recommendations

Sample characteristics	Total sample	Meeting physical activity recommendations	*P*-value
	*n* = 305(%)	*n* = 66 (21.6%)	
BMI (kg/m^2^)			0.600
Normal 18.5–24.99	34 (11)	7 (21)	
Overweight >25–29.99	118 (39)	29 (25)	
Obese >30	153 (50)	30 (20)	
Current medication			
Blood pressure lowering	217 (71)	45 (21)	0.500
Lipid lowering	189 (62)	40 (21)	0.800
Oral-hypoglycaemic drugs	260 (85)	53 (20)	0.200
Insulin	75 (25)	12 (16)	0.200
Diet control	45 (15)	32 (71)	0.200
Duration of diabetes (years)			0.500
< 5	140 (46)	37 (26)	
6 to 11	117 (38)	18 (15)	
12 to 18	33 (11)	6 (18)	
> 18	15 (5)	5 (33)	
Blood pressure (systolic/diastolic) mmHg**			0.500
Within target (<140/<80)	237 (78)	49 (21)	
High (≥140/≥80)	68 (22)	17 (25)	
HbA1c (%)**			0.300
Normal ≤7%	127 (42)	31 (24)	
High >7%	178 (58)	35 (20)	
Fasting lipid profile (mmol/L)**			
Cholesterol Within target (< 5.0)	201 (66)	44 (22)	0.900
Cholesterol High (≥5.0)	104 (34)	22 (21)	
HDL Within target (>1.0)	254 (83)	58 (23)	0.300
HDL Less protective (≤1.0)	51 (17)	8 (16)	
LDL Within target (<2.6)	188 (62)	40 (21)	0.800
LDL High (≥2.6)	117 (38)	26 (22)	
TG Within target (<1.7)	205 (67)	42 (20)	0.500
TG High (≥1.7)	100 (33)	24 (24)	
Comorbidities			0.030*
Yes	277 (91)	55 (20)	
No	28 (9)	11 (39)	

*BMI* body mass index, *HbA1c* Glycated haemoglobin, *HDL* high-density lipoprotein, *LDL* low-density lipoprotein, *TG* triglycerides

*significant *p* < 0.05 based on chi-square analysis

**Oman diabetes mellitus management guidelines (2015)

Over two-thirds of participants (71.0%) were using anti-hypertensive agents, of which most had normal BP readings (77.7%). Sixty-two percent were on statins of which the majority had fasting cholesterol (66.0%), HDL (83.0%), LDL (62.0%) and TG (67.0%) within recommended levels (as per the Oman diabetes management guidelines) [21]. Just over half the sample (58.4%) were found to have uncontrolled diabetes with HbA1c >7%. Compared to males, there were significantly more females with uncontrolled diabetes (61.0% vs 55.0%). Only 9.2% of the total sample were registered with no comorbidities in the clinical notes (Table 2). There was no significant difference in meeting PA recommendations across the physiological variables except for individuals reporting no-comorbidities *P* = 0.030.

### Perceptions on stages and status of physical activity

Eighty-nine percent of the sample reported that PA is important in diabetes management, however the majority (83.0%) reported pre-action stages of PA; the highest proportion considering themselves “not ready” (36.7%). More males than females reported being at an “action” or “maintenance” stage of PA (7.8% vs 2.3%, and 14.0% vs 11.0%, respectively). However, the association of gender with self-reported stages of PA was not statistically significant. Despite 80.0% of the sample reporting that they received PA advice in their respective diabetes clinics, only half of them perceived that they performed sufficient PA/wk. (49.0%) (Table 3). Meeting PA recommendations was higher in individuals reporting being at “Action” stage of PA *P* < 0.001, and/or reporting no barriers to PA.

**Table 3:** Sample characteristics (perceptions on stages and status of PA) and prevalence of meeting physical activity recommendations

Sample characteristics	Total sample	Meeting physical activity recommendations	Not Meeting physical activity recommendations	*P*-value
	*n* = 305(%)	*n* = 66 (21.6%)	*n* = 239 (76.4%)	
Self-reported stages of change in physical activity				<0.001*
Not ready (Pre-contemplation)	112 (37)	8 (7)	104 (93)	
Getting ready (contemplation)	95 (31)	24 (25)	71 (75)	
Preparation	46 (15)	14 (30)	32 (70)	
Action	14 (5)	7 (50)	7 (50)	
Maintenance	38 (12)	13 (34)	25 (66)	
PA advice				0.200
Yes	245 (80)	49 (20)	196 (80)	
No	60 (20)	17 (28)	43 (72)	
Reporting performing sufficient PA/wk				0.050
Yes	150 (49)	39 (26)	111 (74)	
No	155 (51)	27 (17)	128 (83)	
Reporting barriers to performing PA				<0.001*
Yes	177 (58)	24 (14)	153 (87)	
No	128 (42)	42 (33)	86 (67)	
Mean sitting time (SD) min/d	688.1 (143.5)	637.4 (141.2)	702.0 (141.3)	<0.001**
Median sitting time (25th, 75th percentiles) min/d)	705 (600, 780)	600 (540, 720)	720 (600, 840)	

*significant *p* < 0.05 based on chi-square analysis

**non parametric test (Mann-Whitney U test)

Median (25th, 75th percentiles) sitting time was 705 (600, 780) min/d. Individuals meeting PA recommendation had significantly lower sitting time of 600 (540, 720) min/d than 720 (600, 840) min/d in individuals not meeting the recommendation.

### Physical activity and sitting time (GPAQ results)

**Fig. 1:**
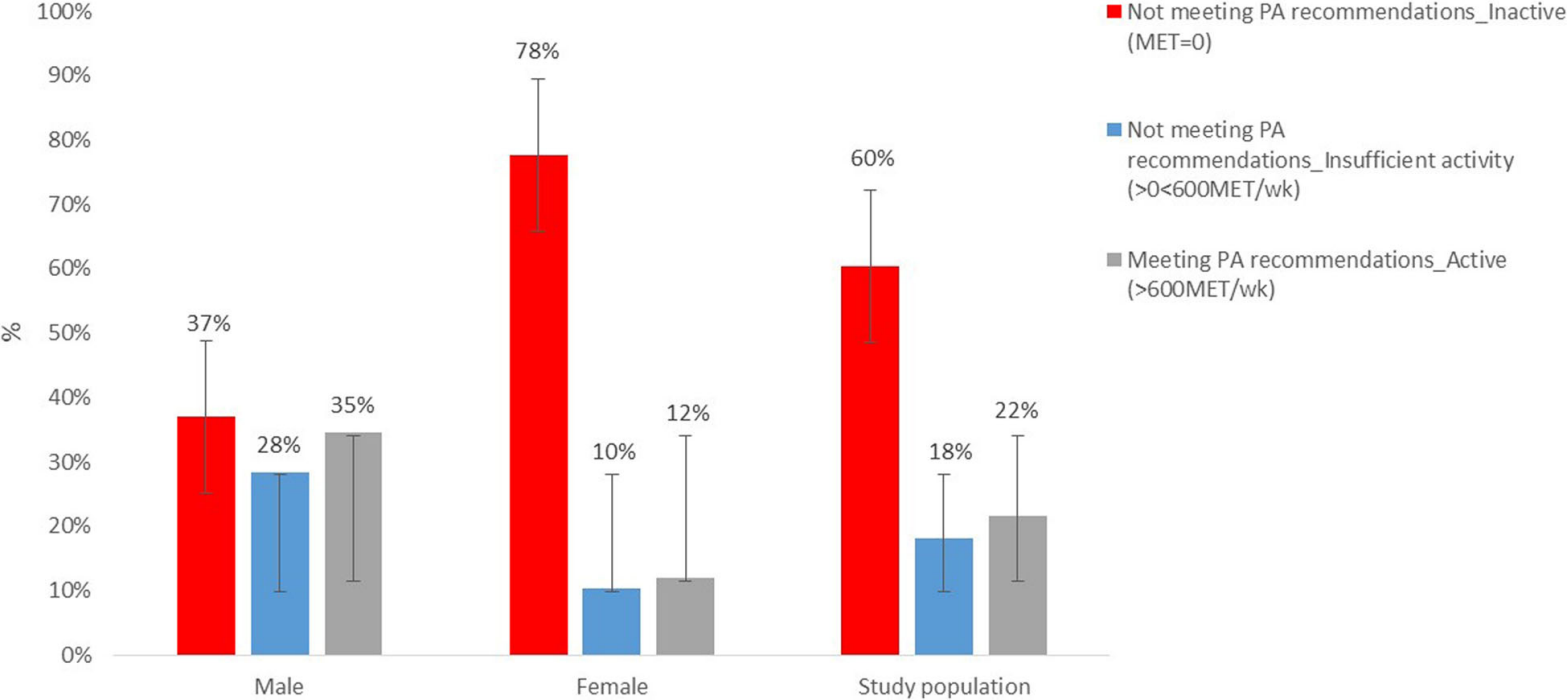
Meeting WHO physical activity recommendations in Omani adults with type 2 diabetes by gender (error bars equals standard error)

Overall, one fifth (21.6%, *n* = 66) of the study population met the recommended WHO PA levels of ≥600 MET-min/wk. (34.6% males vs 12.0% females). The mean (SD) and median (25th, 75th percentiles) MET-min/wk. count achieved was 680 (2347) and 0 (0, 420) min/wk. Mean (SD) and median (25th, 75th percentiles) MET-min/wk. value for individuals meeting the recommendations was 2882 (4405) and 1680 (960, 2790) min/wk., vs 73 (145) and 0 (0, 0) MET-min/wk. for individuals not meeting them. Not meeting PA recommendations was classified as insufficient activity (MET-min/wk. >0 and <600) in 18.0% (*n* = 55) of the population (28.5% males vs 10.3% females) and no activity (MET-min/wk. = 0) in 60.3% (*n* = 184) of the population (36.9% males vs 77.7% females) (Fig. 1).

**Fig. 2:**
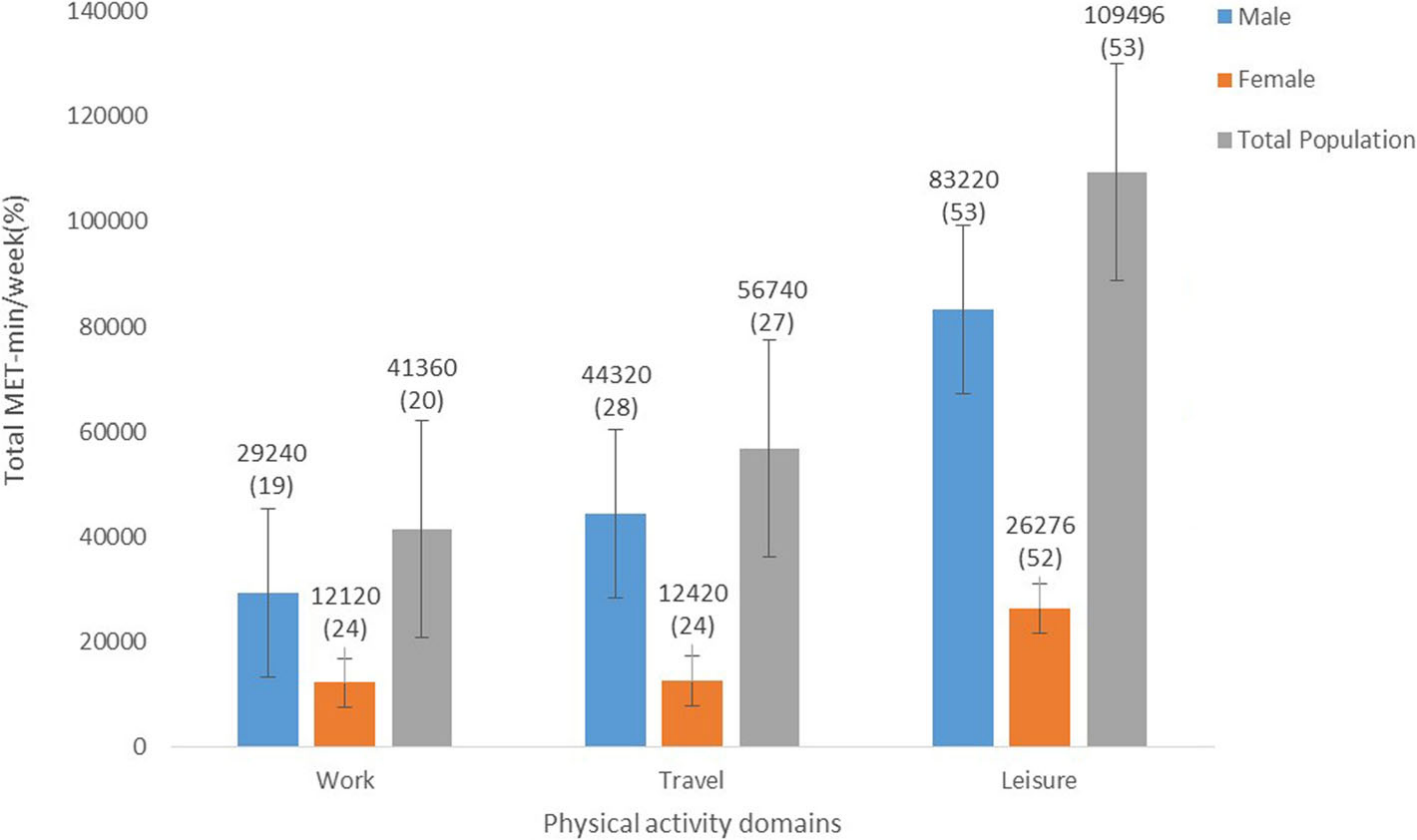
Distribution of total MET-min count (%) across the activity domains (work, travel, and leisure) by gender

Just above half of the total MET-min/wk. from all three domains (207,596 MET-min) was achieved through the ‘leisure’ domain (109,496 MET-min). This was equally true for both males and females, as illustrated in Fig. 2. Compared to males, females were less physically active across the three PA domains (work, travel and leisure).

Binary regression analysis showed that the odds of meeting PA recommendations was higher in males compared to females (OR 4.8, 95% CI 2.5–9.1), in individuals ≤57 years old compared to individuals >57 years old (OR 3.0, 95% CI 1.6–5.9), in individuals reporting ‘active stages’ of PA compared to those ‘not active’ or ‘getting ready’ for PA (OR 2.2, 95% CI 1.2–4.1) and in those who reported no barriers to performing PA compared to those who reported barriers (OR 2.7, 95% CI 1.4–4.9) (Table 4).

**Table 4:** Correlates of meeting WHO PA recommendations in adults with type 2 diabetes

Parameter *n* = 305 (%)	Meeting physical activity recommendations (%)	Not meeting physical activity recommendations (%)	OR	95% CI	Sig
Gender:					
Males = 130 (42.6%)	45 (35)	85 (65)	4.8	2.5–9.1	<0.001
Females = 175 (57.4%)	21 (12)	154 (88)	Ref	.	.
Age:					
≤ 57 = 155 (50.8)	45 (29)	110 (71)	3.0	1.6–5.9	0.001
> 57 = 150 (49.2)	21 (14)	129 (86)	Ref	.	.
Self-reported stages of PA					
Not/getting ready (inactive) = 207 (67.9)	32 (15)	175 (85)	2.2	1.2–4.1	0.009
Preparation/action/maintenance (active) = 98 (32.1)	34 (35)	64 (65)	Ref	.	.
Reporting barriers to performing PA					
No Barriers = 128 (42)	42 (33)	86 (67)	2.7	1.4–4.9	0.002
Reported Barriers = 177 (58)	24 (14)	153 (86)	Ref	.	.

Looking at domain specific correlates of meeting PA recommendations, for the work domain, meeting recommendations was more likely in those reporting they were in ‘active stages’ of PA (OR 4.8, 95% CI 1.4–15.8) and reporting no barriers to PA (OR 4.4, 95% CI 1.2–16.5). Males (OR 9.2, 95% CI 3.2–5.9), individuals ≤57 years (OR 3.1, 95% CI 1.3–7.6) and reporting no barriers to PA (OR 2.5, 95% CI 1.1–5.8) were more likely to meet PA recommendations in travel domain. Males, individuals ≤57 years and those reporting active stages of PA were more likely to meet PA recommendation in the leisure domain (OR 3.1, 95% CI 1.4–6.6, OR 3.1, 95% CI 1.4–7.1 and OR 5.5, 95% CI 2.5–12.0, respectively) (explanatory table is attached in the Additional file 1).

#### Correlates of sitting time

Sitting time ranged from 240 to 890 min/d (4–15 h). Females reported longer sitting time than males. Median (25th, 75th percentiles) sitting time in females was 720 (600, 780) min/d vs 660 (600, 840) min/d in males. However, gender was not significantly associated with prolonged sitting *P* > 0.050. Age on the other hand, was the only significant correlate for longer sitting time. Older individuals (>57 years) had significantly longer sitting time compared to individuals ≤57 years (OR 2.8, 95% CI 1.7–4.6).

#### Preferences for PA and intervention delivery components

When participants were asked to select their preferred PA for which they would like to get support, walking was of interest to 97.4% of the study population. Just over a third of the sample (38.0%) were interested in PA consultations/clinics integrated in routine diabetes care in primary care setting followed by structured PA exercises (13.0%) and PA referrals (6.0%). Whilst 27.0% suggested mixed PA components including consultations/clinics, structured exercises, and referrals to PA facilities, other participant, reported “Don’t know” and “no preferred PA component” (12.0% and 4.0% respectively).

Less than half of the sample participants reported they “did not know” who should be responsible for PA in diabetes care (42.0%). The diabetes doctor was selected by a fifth of the population (22.0%) followed by the dietician (9.0%), and 27.0% reported various other healthcare professional namely physiotherapists, PA experts, diabetes nurse and health educator.

## Discussion

The aim of this study was to estimate levels of PA, sitting time and the factors associated with meeting PA recommendations and prolonged sitting time in adults with T2D in Oman. The work reported contributes to limited literature on PA internationally and in particular PA patterns in diabetes care in the GCC.

Worth mentioning, the response rate in this study was high possibly due to collecting data within clinical settings where participants felt comfortable to participate in the study during their waiting time for their routine diabetes clinics.

Evidence on activity levels in populations with T2D is variable across countries. However, low levels of PA in T2D populations have been reported in several studies [15] including those from Arabic speaking countries [16, 22, 23]. Whilst meeting PA recommendations in this study is higher than national levels (15%), activity levels in the current study are much lower than those reported in populations with T2D in the UK (34%) [24] and USA (36 to 50%) [25]. Of greatest concern is the fact that more than half (60.3%) of this study sample, compared to 55% in similar studies, reported no activity (MET = 0) [26], indicating significant inactivity levels. Despite the differences in PA study tools that may contribute to disparities in PA levels across studies, the inactivity levels in this study population is disappointing in view of the consistent evidence on the physiological, metabolic and haemodynamic benefits of PA in the management of T2D [27].

Males, younger age (<57 years), reporting being at “active stages” of PA and reporting “no barriers” to performing PA were significant positive factors associated with meeting PA recommendations in this study sample. The global trend of male dominance in meeting PA recommendation was prominent in the travel domain followed by the leisure domain. Higher travel activity levels in males could be due to cultural and religious factors in Arabic and Muslim countries. Congregational prayers in mosques are considered to have more social and spiritual benefit than praying by oneself. Males value being able to walk to and from the mosques five times every day for their daily prayers especially given that every neighbourhood has access to mosques [28]. Females, who are more likely to be obese [29], prefer to pray and stay at home for child care reasons. This may additionally be augmented by a lack of gender specific facilities and safe places for females to perform PA activity as reported in neighbour countries namely UAE and Saudi Arabia [30–32]. Hence gender segregated PA promotional interventions for adults with T2D should target females who are more vulnerable to inactive behaviour and uncontrolled diabetes. On the contrary, a study in Lebanon reported that females were more active in both the general population, as well as the population with T2D [16]. The difference in gender effect might be attributed to PA supportive cultural, educational, environmental and economic status specific to Lebanon that requires further exploration to learn lessons on effective PA interventions for females that could possibly be tested in Oman.

Despite the higher absolute leisure activity in males compared to females, the relative amounts of leisure activity contributed the most to the overall activity levels, in both males and to females. Leisure time PA has been reported to be significantly associated with reduced mortality risks (20% to >37%) and favourable cardiovascular outcomes [33]. No clear association or even an inverse relationship is observed for work or travel PA [34]. In general, individuals who had not met PA recommendations in this study had higher blood pressure, HbA1c, lipid profile, and more comorbidities. Hence, PA promotional interventions should consider opportunities within activity domains: work, travel and most importantly leisure for sub-populations with T2D across the various cultures. Meeting PA recommendations in travel and leisure domains was also seen to be more likely in younger individuals. Younger individuals in the current study have less comorbidities and hence may experience less discomfort compared to older aged individuals with T2D who might be concerned about their disease condition [15].

In the current study, self-reported PA stages of change namely “pre-contemplation” and “contemplation” was associated with low activity levels specifically in work and leisure domains. The fact that more than half of the study population were at in-active stages of PA raises concerns in view of the current diabetes care in Oman that specifies the provision of advice on PA [21]. This is a critical finding as the majority (80.0%) of the study population indicated that they received PA advice, but this was not associated with being physically active or meeting PA recommendations. The current PA advice practiced in routine diabetes care should include behaviour change techniques to ensure stage progression for individuals with T2D from pre-action to action and maintenance stages of change. In a recent study, five behaviour change techniques, namely prompt focus on past success, barrier identification/problem-solving, use of follow-up prompts, provide information on where and when to perform the behaviour and prompt review of behavioural goals of PA were significantly associated with increased PA behaviour in T2D and improving HbA1c [35]. Practicing PA barrier identification across activity domains is important as responding “yes” to barriers to performing PA in the current study was apparent in those not meeting the PA recommendations specifically in work and travel activity domains. Hence, opportunities for culturally suitable active workplaces and transportation should be identified and considered.

Despite using the same measurement tool, the average sitting time in this in this study population with T2D was almost six times higher than what has been reported locally in the Sur general population of 120 min/d [11]. This disparity could be attributed to differences characteristics and disease condition of the studied population. Similarly, the average sitting time in the current study was more than double the time in adults with T2D in Canada of 278 min/d [14], however different measuring tool was used. Given the evidence on the increased risk of cardio-vascular mortality with long sitting time on health [36], PA interventions should emphasise shorter and interrupted sitting time especially for vulnerable sub-groups with T2D. However, further research for the population with T2D on domain-specific sedentary behaviours is necessary to plan for appropriate public health interventions targeting more PA and less sedentary behaviour.

The only correlates for longer sitting time in this population study was older age (>57 years). This finding corresponds to a study in the USA that reported increase in sedentary time with age for both men and women in the general population [37]. However, gender stratified significant correlates of longer sitting time in the study in Sur were younger age, employed individuals, higher BMI (in females) and higher education (in males). However, lack of significant associations of long sitting time with any other socio-economical or clinical variables in populations with T2D was evident in a study in Canada where only being a non-immigrant, and having a university degree were the factors associated with more min/d spent sitting [38]. Variations in significant correlates can be attributed to differences in definitions of sedentary behaviour including insufficient PA, and sitting time and differences in measurement tools [39].

Similar to a study in Scotland in adults with T2D [24], walking was the preferred activity over running, cycling, swimming and other activities indicated by this population. Walking interventions combined with pedometers as motivational tools are more likely to improve PA behaviour in the general population and adults with T2D [40, 41]. Hence, irrespective of culture, walking can be considered as an appropriate method of PA promotion for adults with T2D as they are sedentary individuals and aware of their need for lifestyle change.

In the current study population, just over a third were interested in PA consultations in routine diabetes care. In terms of who participants felt they would prefer to be responsible in delivering PA services within diabetes care, 40% selected “don’t know” while only a fifth (22%) preferred diabetes doctors. The fact that participants were unsure on the health worker for PA promotion opens an opportunity to utilize other “non-doctors” health care providers to endorse PA within diabetes care. Effectiveness of PA consultations linked to behaviour change techniques in increasing PA behaviour in population with diabetes has been consistent in several reviews and randomized control trials carried out in the UK, Canada, USA and Belgium [35, 42]. This approach is yet to be investigated in the Arab world.

The findings of the current study are considered in light of several limitations. Due to the cross-sectional nature of the study, the associations reported may not indicate causality. One must acknowledge potential errors associated with self-reported measures of PA and sitting time. The subjective nature of PA self-reporting measurement tools used across studies may have contributed to discrepancies in PA recordings especially when PA definitions (moderate and vigorous) are explained differently. In general self-reporting PA measurement tools do not provide accurate estimates (limited validity and reliability) especially across distinct demographic, cultural groups [43]. Moreover, PA questionnaires, namely GPAQ in this study, are less sensitive to quantifying mild daily activities that are reported to be the major activity in older and sub-populations [44]. Validating GPAQ for this population using an objective measure may be useful for quantifying activity levels and ultimately effective PA promotional interventions.

## Conclusions

Overall, levels of PA were low across all activity domains and median sitting time was high. Females, older age, reporting ‘in-active stages’ of PA and barriers to PA were negatively correlated with meeting PA recommendations. Given the significant association of meeting PA recommendations with gender, interventions to modify PA behaviours should be linked to gender-specific barriers to PA. Sitting time in older individuals with T2D was greater than regional and global estimates. PA consultations based on behaviour change techniques and which are specific to individual PA stages of change may be promising strategies to increasing PA behaviour and reduce sitting time.

## Additional file

Additional file 1: Table correlates of physical activity across domains. (PDF 128 kb)

## Abbreviations

BMI: Body mass index
CI: Confidence interval
GCC: Gulf Cooperation Council
GPAQ: Global physical activity questionnaire
HDL: High density lipoproteins
LDL: low density lipoproteins
OR: odds ratio
PA: Physical activity
SD: Standard deviation
SE: Standard error
T2D: Type 2 diabetes
TG: Triglycerides
WHO: World Health Organization
