# Supplementary material for: Correlates of physical activity and sitting time in adults with type 2 diabetes attending primary health care in Oman
Source: BMC Public Health. 2017 Aug 1;18:85. doi: 10.1186/s12889-017-4643-7 (PMC5539993; doi:10.1186/s12889-017-4643-7)
Supplement: Additional file 1: — Table correlates of physical activity across domains. (PDF 128 kb) [file 12889_2017_4643_MOESM1_ESM.pdf]

(%)	Work					Travel					Leisure				
	Meeting 14 (4.6)	Not meeting 291 (95.4)	OR	CI	Sig	Meeting 29 (9.5)	Not meeting 276 (90.5)	OR	CI	Sig.	Meeting 37 (12.1)	Not meeting 268 (87.9)	OR	CI	Sig.
Gender															
Male 130(42.6)	8 (6.2)	122 (93.8)	0.97	0.9-1.0	0.2	24 (18.5)	106 (81.5)	9.2	3.2-25.9	<0.001**	24 (18.5)	106 (81.5)	3.1	1.4-6.6	0.005*
Female 175(57.4)	6 (3.4)	169 (96.6)	Ref	.	.	5 (2.9)	170 (97.1)	Ref	.	.	13 (7.4)	162 (92.6)	Ref	.	.
Age															
≤57 155(50.8)	9 (5.8)	146 (94.2)	0.97	0.9-1.0	0.3	20 (12.9)	135 (87.1)	3.1	1.3-7.6	0.01*	27 (17.4)	128 (82.6)	3.1	1.4-7.1	0.006*
>57 150(49.2)	5 (3.3)	145 (96.7)	Ref	.	.	9 (6.0)	141 (94.0)	Ref	.	.	10 (6.7)	140 (93.3)	Ref	.	.
Self-reported stages of PA															
Preparation/action/maintenance 98(32.1)	10 (10.2)	88 (89.8)	4.8	1.4-15.8	0.01*	9 (9.2)	89 (90.8)	0.9	0.9-1.1	0.9	26 (26.5)	72 (73.5)	5.5	2.5-12.0	<0.001**
Not/getting ready 207(67.9)	4 (1.9)	203 (98.1)	Ref	.	.	20 (9.7)	187 (90.8)	Ref	.	.	11 (5.3)	196 (94.7)	Ref	.	.
Reporting barriers to performing PA															
No barriers 128(42.0)	11 (8.6)	117 (98.3)	4.4	1.2-16.5	0.03*	19 (14.8)	109 (85.2)	2.5	1.1-5.8	0.03*	22 (17.2)	106 (91.5)	0.9	0.9-1.0	0.8
Reported barriers 177(58.0)	4 (1.7)	173 (98.30	Ref	.	.	10 (5.6)	167 (94.4)	Ref	.	.	15 (8.5)	162 (91.5)	Ref	.	.
